# The optimal temperature of air-heated blankets for preventing intraoperative hypothermia in patients with gynecological tumor: a randomized controlled trial

**DOI:** 10.3389/fmed.2026.1835872

**Published:** 2026-08-05

**Authors:** Dan Jin, Pan Youxuan, Yang Li, Shuxian Zhang

**Affiliations:** 1 Department of Operating Center, Women and Children's Hospital of Jiaxing University, Jiaxing, China; 2 Department of Surgery and Anesthesiology, Women and Children's Hospital of Jiaxing University, Jiaxing, China

**Keywords:** air-heated blanket, complications, gynecological surgery, hypothermia, tumor

## Abstract

**Background:**

Air-heated blankets can effectively prevent intraoperative hypothermia in patients undergoing major surgeries. However, the optimal temperature of an air-heated blanket for preventing intraoperative hypothermia in these patients has not yet been established. This study aimed to determine the optimal temperature of an air-heated blanket for preventing intraoperative hypothermia in patients undergoing radical resection for endometrial cancer.

**Method:**

After the induction of general anesthesia, groups A, B, and C received air-heated blankets set to 36°C, 38°C, and 40°C, respectively, for warming until the end of surgery. The core temperature, time to eye opening, extubation time, and time to discharge from the post-anesthesia care unit (PACU) were recorded. Perioperative complications—including hypothermia, emergence agitation, hyperthermia, shivering, delayed awakening, and nausea and vomiting—were also documented.

**Results:**

There were six cases of hypothermia in group A, while no hypothermia was observed in groups B and C. Statistically significant differences were found in the incidence of hypothermia and shivering among the three groups (*P* = 0.002 and *P* = 0.034, respectively). The extubation time and time to eye opening in group C were shorter than those in group A (*P* < 0.05); however, no statistically significant differences were observed between groups B and C.

**Conclusion:**

The optimal temperature of the air-heated blanket for preventing hypothermia in patients undergoing radical resection for endometrial cancer was 38°C. This study offers guidance on the specific warming temperatures used in clinical practice.

**Clinical Trial registration:**

Identifier: ChiCTR2400080803, https://www.chictr.org.cn/.

## 1 Introduction

Perioperative hypothermia is common during major surgeries, with an incidence ranging from 20 to 70% (1, 2). Several factors contribute to perioperative hypothermia, including preoperative exposure, low operating room temperatures, skin exposure during preparation, and the use of washing solutions. Additionally, anesthesia suppresses the hypothalamic-mediated physiological response to hypothermia (3). Perioperative hypothermia adversely affects coagulation, increases blood loss, alters drug metabolism, raises the risk of surgical site infections, prolongs hospital stays, and delays discharge from the post-anesthesia care unit (4, 5). Currently, air-heated blankets are widely used to prevent intraoperative hypothermia in patients undergoing major surgeries (6–8). However, the optimal temperature setting for air-heated blankets to prevent intraoperative hypothermia in these patients has not yet been established. Although air-heated blankets typically offer multiple temperature settings, the effectiveness of different temperatures in preventing hypothermia remains unclear. Therefore, this study aimed to determine the optimal temperature of an air-heated blanket for preventing intraoperative hypothermia in patients undergoing radical resection for endometrial cancer, providing a reference for clinical practice.

## 2 Methods

This study was approved by the Ethics Committee of Jiaxing university (approval number: JX2024Y005), and informed consent was obtained from all patients. This trial was registered at the Chinese Clinical Trial Registry (registry number: ChiCTR2400080803). From October 2024 to October 2025, a total of 105 patients with American Society of Anesthesiologists (ASA) physical status II, aged 30–75 years, who underwent radical resection for endometrial cancer were enrolled in this study. The exclusion criteria were as follows: abnormal preoperative temperature (above 37.5°C or below 36.0°C), expected anesthesia duration of less than 2 h, presence of acute infectious diseases, and hyperthyroidism.

### 2.1 Anesthesia method

Prior to the study, a randomization sequence for 105 codes divided into three equal-sized groups was generated using an online randomization generator. These codes were placed into a sealed envelope by a nurse who was not involved in this study. The investigators, anesthesiologists, surgeons, and patients were blinded to grouping.

After the patient entered the operating room, the room temperature was set at 24°C, and the humidity was maintained between 40 and 60%. Blood pressure (BP), electrocardiogram (ECG), pulse oxygen saturation (SpO_2_), and heart rate (HR) were monitored. A peripheral venous catheter was inserted, and Ringer's solution was infused intravenously at a rate of 6–8 ml/kg/h. General anesthesia was induced with intravenous midazolam 0.03 mg/kg, propofol 2 mg/kg, sufentanil 0.4 μg/kg, and cisatracurium 0.15 mg/kg. Mechanical ventilation was initiated following endotracheal intubation under direct laryngoscopic visualization, with a respiratory rate of 10–12 breaths per min, a tidal volume of 8 mL/kg, and an inspiratory-to-expiratory ratio (I: E) of 1:2. Propofol and remifentanil were administered as separate infusions to maintain intraoperative systolic blood pressure within 20% of the baseline value by adjusting their infusion rates. The depth of anesthesia was maintained with BIS values between 40 and 55. At the end of the operation, the patient was transferred to the post-anesthesia care unit (PACU). If the intraoperative systolic blood pressure was lower than 90 mmHg, ephedrine 6 mg was administered by intravenous injection. If the heart rate was less than 50 beats/min, atropine 0.3 mg was administered intravenously.

### 2.2 Intervention and measurement

On the basis of routine heat preservation nursing measures, all patients were warmed using an air-heated blanket (Maikang Medical Company, Beijing, China), with the temperature set at 36°C, 38°C, and 40°C, respectively. The air-heated blanket was placed underneath the patient and was turned on after anesthesia was induced, remaining in use until the end of the operation. The pre-anesthesia body temperature (tympanic temperature) was measured using an infrared ear thermometer. Intra-anesthesia body temperature (nasopharyngeal temperature) was continuously monitored with a temperature probe, and post-anesthesia body temperature (tympanic temperature) was again measured using an infrared ear thermometer. Air-heated blankets were suspended if body temperature was higher than 37.5°C. Air-heated blankets were set to 40°C to restore normal body temperature as quickly as possible if the intraoperative temperature fell below 36°C. Then the appropriate temperature (40.0°C or 38°C) was selected.

The core body temperature was recorded immediately after anesthesia, at 30 min, 1, 1.5, and 2 h after anesthesia, and at the end of the surgery. Intraoperative blood loss, time to eye opening, extubation time, and time to discharge from PACU were recorded. Perioperative complications such as hypothermia, emergence agitation, delayed awakening, hyperthermia, shivering, nausea, and vomiting were also noted. Intraoperative hypothermia was defined as a core body temperature < 36.0°C. Hyperthermia was defined as a core body temperature ≥37.5°C. Tramadol 50 mg was administered intravenously if postoperative shivering occurred, and ondansetron 4 mg was given intravenously if nausea and vomiting developed. The criteria of extubation were as follows: spontaneous breathing with tidal volume > 6 ml/kg, respiratory rate > 10 breaths/min, and SpO_2_ > 96%. The standard for discharge from the PACU was an Aldrete score of ≥ 9. Delayed awakening was defined as the inability to open the eyes within 2 h after the end of the operation.

### 2.3 Sample size

The primary outcome was hypothermia, and the secondary outcomes were the time to eye opening and extubation time. The sample size was calculated using PASS^®^ version 11.0.7 (NCSS, LLC, Kaysville, UT, USA). Based on a preliminary test with 20 samples each group showed that the proportions of hypothermia were 0%, 0%, and 25%, respectively. A sample size of 29 patients was needed to detect a statistical difference among the 3 groups with an α error of 0.05 and a power of 0.8. The sample size was increased to 35 to account for dropout. Thus, a total of 105 patients (35 cases per group) were required.

### 2.4 Statistical analysis

Statistical analyses were performed using SPSS 22.0 software. Measured data with a normal distribution were expressed as the mean ± standard deviation, and one-way analysis of variance was used for comparisons; the K-W test was used for non-normally distributed data. The χ^2^ test or Fisher's exact test was used to determine the ratio of count data. Statistical significance was set at *P* < 0.05.

## 3 Results

**Figure 1:**
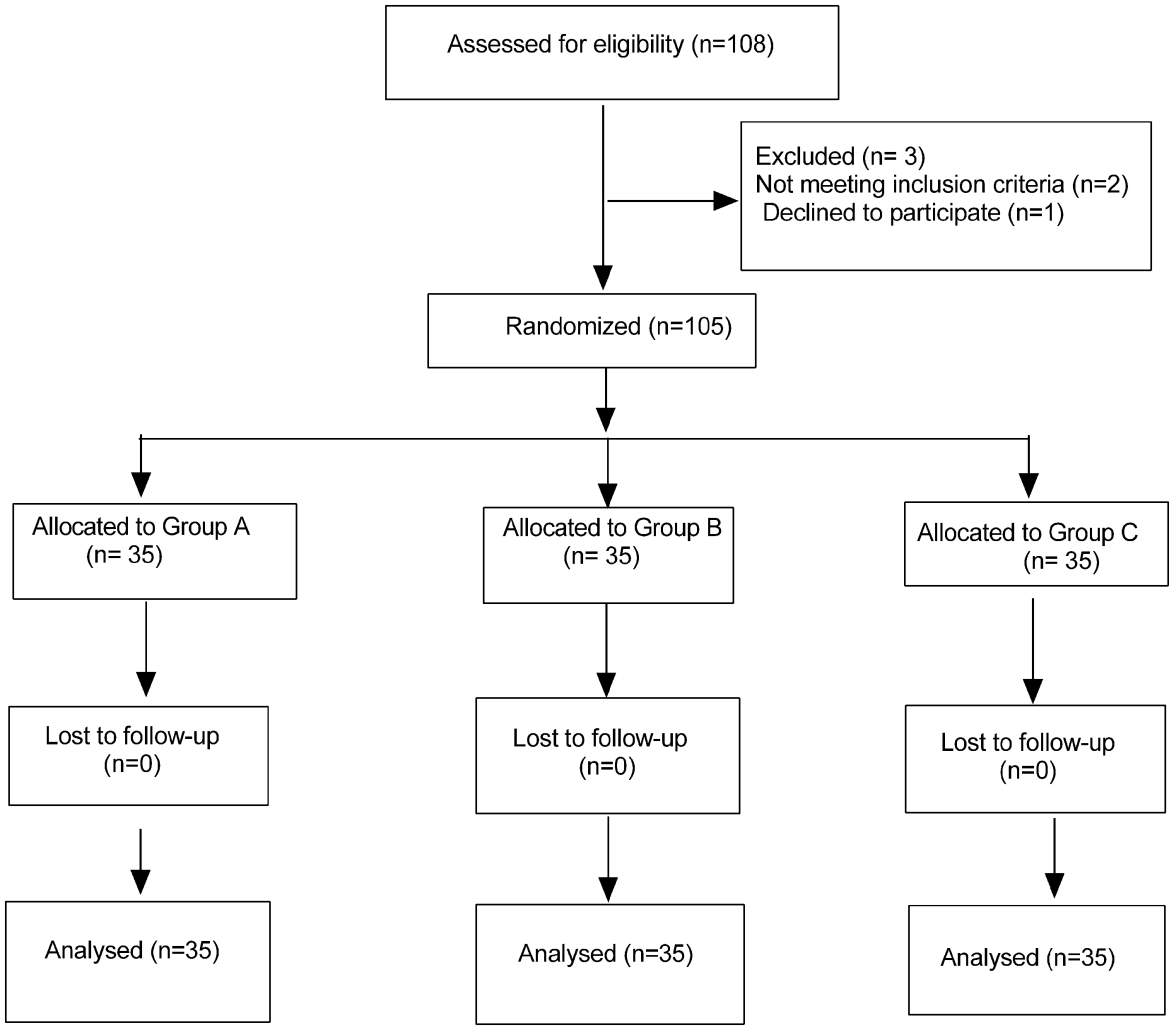
Flow diagram of the study.

Initially, 108 patients were enrolled in this trial; Finally, 105 patients were randomly assigned to Groups A, B and C (*n* = 35). The flow diagram of this study is shown in Figure 1. There were no statistically significant differences in age, ASA status, weight, height, duration of anesthesia or intraoperative blood loss among the 3 groups (*P* > 0.05, Table 1).

**Table 1:** Comparison of general data among the 3 groups (*n* = 35).

Index	Group A	Group B	Group C	*P*-value
Age (year)	53.5 ± 10.2	55.8 ± 8.8	52.7 ± 7.7	0.339
Weight (kg)	57.8 ± 7.5	59.5 ± 9.0	58.3 ± 6.0	0.610
Height (cm)	160.7 ± 3.8	159.7 ± 3.0	160.6 ± 3.7	0.381
ASA (I/II)	4/31	3/32	5/30	0.754
Duration of anesthesia (min)	171.7 ± 37.8	167.9 ± 37.1	169.2 ± 40.2	0.915
Amount of infusion (ml)	1,535.7 ± 50.5	1,522.3 ± 61.4	1,508.6 ± 60.1	0.148
Blood loss (ml)	152.0 ± 33.6	161.4 ± 46.0	148.0 ± 30.2	0.305
Extubation time (min)	14.3 ± 2.2	12.9 ± 2.1	12.7 ± 2.4	0.006^*^
Time to eye opening (min)	19.3 ± 5.3	17.1 ± 4.4	16.8 ± 3.5	0.042^*^
Time to discharge from PACU (min)	37.1 ± 5.0	36.8 ± 5.7	34.8 ± 4.2	0.128

Data are presented as mean ± SD or numbers. ^*^P < 0.05

### 3.1 Core temperature

**Figure 2:**
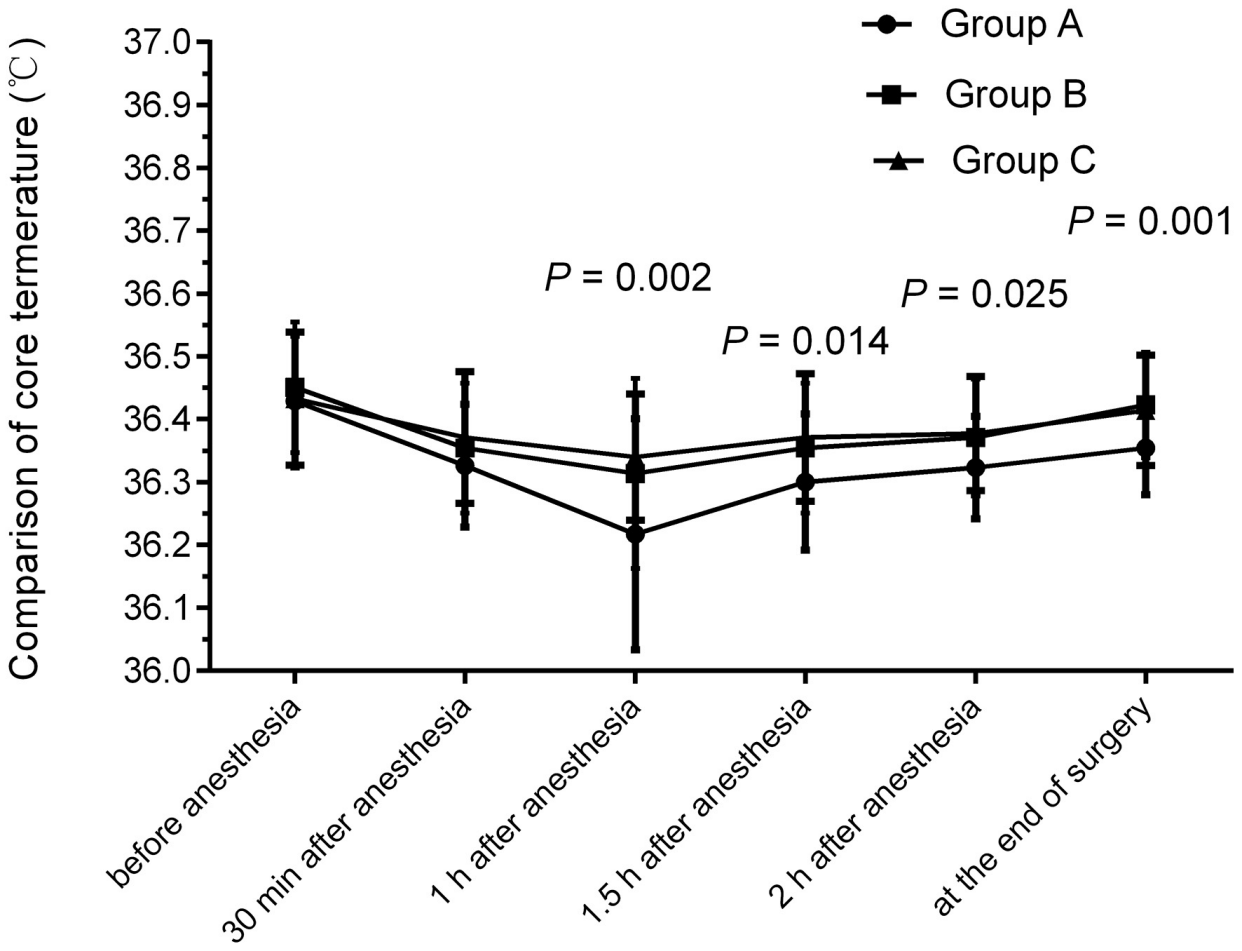
Comparison of the core body temperature.

There were statistically significant differences in core temperature among the three groups from 1 h after anesthesia to the end of surgery. The core temperature of groups B and C was significantly higher than that in group A from 1 h after anesthesia to the end of surgery (*P* < 0.05), but there were no statistically significant differences at other time points. There were not statistically significant differences between the groups B and C at any time points (*P* > 0.05, in Figure 2).

### 3.2 Recovery time

There were statistically significant differences in the extubation and time to eye opening among the three groups, but there were no statistically significant differences in time to discharge from PACU among the three groups. The extubation time and time to eye opening were significantly shorter in groups B and C than in group A (*P* < 0.05), but there was no statistically significant difference between groups B and C (Table 1).

### 3.3 Adverse events

There was statistically significant difference in the incidence of hypothermia and shivering among the three groups (*P* = 0.002 and *P* = 0.034, respectively), but there were no statistically significant differences in other complications such as emergence agitation, delayed awakening, hyperthermia, nausea, and vomiting, incision infection and scalding (*P* > 0.05). There was no statistically significant difference in the incidence of hypothermia, shivering, emergence agitation, hyperthermia, delayed awakening, nausea and vomiting, incision infection, or scalding between groups B and C (Table 2).

**Table 2:** Adverse events of perioperative period among the 3 groups (*n* = 35).

Index	Group A	Group B	Group C	*P*-value
Hypothermia (*n*)	6	0	0	0.002^*^
Emergence agitation (*n*)	3	1	1	0.457
Nausea and vomiting (*n*)	2	1	1	0.782
Respiratory depression (*n*)	2	1	1	0.782
Shivering (*n*)	3	0	0	0.034^*^
Delayed awaking (*n*)	1	0	0	0.107
Incision infection (*n*)	0	0	0	/
Scalding (*n*)	0	0	0	/
Hyperthermia (*n*)	0	0	1	0.107

Data were presented as numbers. ^*^P < 0.05

## 4 Discussion

This study found that in patients undergoing radical resection for endometrial cancer, the optimal temperature of the air-heated blanket to prevent intraoperative hypothermia was 38°C, which was associated with a lower incidence of postoperative complications.

This study found that patients who used air-heated blankets set to 38°C or 40°C did not develop hypothermia after anesthesia, whereas six patients in the 36°C group did develop hypothermia. The patients' body temperatures were similar when the air-heated blankets were set to 38°C or 40°C, but these temperatures were significantly higher than those in the 36°C group. These findings demonstrated that patients using air-heated blankets set to 36°C still experienced hypothermia, while those using blankets set to 38° or 40°C were effectively protected against it. The air-heated blanket delivers high-convection hot gas to the surface of the body, with insulation becoming more effective at higher temperatures. After anesthesia, the patient's temperature gradually decreases due to vasodilation and suppression of the thermoregulatory center by anesthetic drugs. Additionally, intraoperative opening of the body cavity and the use of low-temperature disinfectants and flushing fluids increase heat loss, leading to a further drop in body temperature (1). Currently, air-heated blankets are considered an effective heating measure (6, 7). Active heating of the air-heated blanket mainly transmits heat directly to the skin and surrounding tissues to maintain the body temperature within the normal range. This study showed that air-heated blankets set to 38°C were sufficient to maintain intraoperative temperature stability and prevent the occurrence of intraoperative hypothermia. A randomized controlled study demonstrated that using an air-forced blanket set to 38°C was an effective method for preventing hypothermia in elderly patients undergoing transurethral resection of the prostate (9). He et al. (10) found that air-heated blankets set at 38°C maintained stable body temperatures with fewer fever in pediatric patients undergoing surgery for developmental displacement of the hip, compared with air-heated blankets set at 32° and 43°C. These findings were consistent with our results. Many studies have shown that air-heated blankets can effectively prevent hypothermia during operation. However, most studies do not specify the exact warming temperature when using the air-heated blanket, instead categorizing the application temperature as “high,” “medium,” or “low” depending on the device.

In this study, the extubation and eye-opening times were shorter in groups B and C than in group A. Temperature influences the metabolism of anesthetics; within a certain range, an increase in temperature accelerates anesthetic metabolism, leading to faster recovery. Zhang et al. (11) found that using an air-heated blanket reduced the incidence of hypothermia and shortened recovery time in elderly patients undergoing laparoscopic radical resection of colorectal cancer, which was consistent with the findings of this study.

In this study, the incidence of hypothermia and shivering was reduced in groups B and C. This indicated that higher temperatures could maintain normothermia. The occurrence of shivering is influenced by several factors. Previous studies have suggested that anxiety, young age, and hypothermia may be associated with intraoperative shivering (12, 13). Perioperative hypothermia affects various body systems. Hypothermia leads to decreased thrombin activity and prolonged bleeding time, thereby causing increased intraoperative blood loss (4). No significant differences were observed in the incidence of emergence agitation, nausea and vomiting, hyperthermia, delayed awakening, incision infection or scalding among the three groups in this study, which may be related to the small sample size. There are many reasons for emergence agitation following general anesthesia, with pain and discomfort being the main causes of postoperative agitation. Literature demonstrated that maintaining normothermia intraoperatively decreased the incidence of infectious complications in patients undergoing colorectal resection. Perioperative hypothermia promotes surgical-wound infection by triggering thermoregulatory vasoconstriction (4). Although no scalding occurred in the 40°C temperature mode, concerns remain worries. Chung et al. reported a case involving a 37-year-old woman who sustained a burn injury due to the misuse of a forced-air warming device set at a temperature of 43°C (14). The potential side effects of exposure to 40°C (e.g., skin burns, discomfort) should not be overlooked in clinical practice.

**Limitations** of this study include that it is a single-center, small-sample study; patient age, gender, and operation time may affect the study results; and the effect of the air-heated blanket on the body temperature of patients undergoing other types of surgery needs further study.

## 5 Conclusion

The optimal temperature of the air-heated blanket for preventing hypothermia in patients undergoing radical resection for endometrial cancer is 38°C. This study offers guidance on the specific warming temperatures used in clinical practice.

## Data Availability

The original contributions presented in the study are included in the article/supplementary material, further inquiries can be directed to the corresponding author.
